# Evaluation of a Non-Enzymatic Electrochemical Sensor Based on Co(OH)_2_-Functionalized Carbon Nanotubes for Glucose Detection

**DOI:** 10.3390/s24237707

**Published:** 2024-12-02

**Authors:** Diego Bolaños-Mendez, Lenys Fernández, Rafael Uribe, Alisson Cunalata-Castro, Gema González, Isamara Rojas, Andrés Chico-Proano, Alexis Debut, Luis Alberto Celi, Patricio Espinoza-Montero

**Affiliations:** 1 Escuela de Ciencias Químicas, Pontificia Universidad Católica del Ecuador, Quito 170525, Ecuador; dgbolanos@puce.edu.ec (D.B.-M.); amcunalatac@puce.edu.ec (A.C.-C.); 2 Departamento de Ingeniería Química, Escuela Politécnica Nacional, Quito 170525, Ecuador; rfael.uribe@epn.edu.ec (R.U.); isamara.rojas@epn.edu.ec (I.R.); andres.chico@epn.edu.ec (A.C.-P.); alberto.celi@epn.edu.ec (L.A.C.); 3 Escuela de Ciencias Físicas y Nanotecnología, Universidad Yachay Tech, Urcuqui 100650, Ecuador; ggonzalez@acta-microscopica.org; 4 Centro de Nanociencia y Nanotecnología, Universidad de las Fuerzas Armadas ESPE, Sangolqui 170501, Ecuador; apdebut@espe.edu.ec

**Keywords:** carbon nanotubes, cobalt hydroxide, glucose, electrochemical sensors, non-enzymatic

## Abstract

This work reports on the assessment of a non-hydrolytic electrochemical sensor for glucose sensing that is developed using functionalized carbon nanotubes (fCNTs)/Co(OH)_2_. The morphology of the nanocomposite was investigated by scanning electron microscopy, which revealed that the CNTs interacted with Co(OH)_2_. This content formed a nanocomposite that improved the electrochemical characterizations of the electrode, including the electrochemical active surface area and capacitance, thus improving sensitivity to glucose. In the electrochemical characterization by cyclic voltammetry and chronoamperometry, the increase in catalytic activity by Co(OH)_2_ improved the stability and reproducibility of the glucose sensor without the use of enzymes, and its concentration range was between 50 and 700 μmol L^−1^. The sensor exhibited good linearity towards glucose with LOD value of 43.200 µmol L^−1^, which proved that the Co(OH)_2_-fCNTs composite is judicious for constructing cost effective and feasible sensor for glucose detection.

## 1. Introduction

There are about 420 million diabetes patients around the world, and it is estimated that this will increase up to 642 million by the year 2040. This alarming trend highlights the immediate necessity of developing more sensitive, stable, and even miniaturizable glucose sensors for continuous blood glucose monitoring (CGM) use and hypoglycemia management [1,2]. Recently, the use of electrochemical sensors has gained relevance over conventional ones for the analysis and detection of various analytes in different areas, such as environmental monitoring [3,4], disease [5], and food [6,7,8]. Currently, and specifically in the case of glucose, glucose oxidase (GOx)-based enzymatic sensors have been established, broadly due to their high selectivity and sensitivity towards glucose [2,9,10,11]. Temperature and pH can interfere with the proper functioning of these sensors, making them sensitive to environmental conditions [12,13], which raises their cost compared to the original sensors as they exhibit poor stability. Moreover, the cumbersome enzyme immobilization operation limits their industrial application [14].

Under such circumstances, non-enzymatic electrochemical sensors have been under high consideration as the best substitute [12,13,14,15,16,17,18,19,20,21,22].

Non-enzymatic sensors eliminate many of the aforementioned shortcomings, indicating higher stability, lower costs, and increased protection from negative effects of environmental conditions. These sensors measure the direct electro-oxidation of glucose on the surface of the electrodes using of new materials including noble metals and their alloys, transition metals, metal oxides, and nanomaterials with the objective of improving the catalytic properties of the electrochemical process [14,15,16,18,19,20,22]. Recent developments of electrode materials have hit the mark regarding both the selectivity and sensitivity towards the target analyte and these electrodes are employed in portable devices especially for the continuous monitoring of glucose levels [12,19].

Electrochemical sensors have recently attracted significant attention for the incorporation of nanomaterials. Carbon nanotubes (CNTs) are the most commonly used nanomaterials for heuristic application due to their thermal, elastic, and electric properties enhancing the analytical characteristics of sensors. Also, CNTs as conductive support are used in the electrode modification with different metal composites and the chemical modification of the electrode surface by metals improves adsorption capacity, biocompatibility, and catalytic activity for the definite analyte [11,23,24,25,26,27,28,29]. Manganese, copper, nickel, platinum, and cobalt are among the groups of transition metals that have been employed in the non-enzymatic detection of glucose based on their excellent electrocatalytic activity and high electron transfer. Of the aforementioned materials, cobalt and its derivatives, including cobalt hydroxide (Co(OH)_2_), have been widely used, mainly due to their high sensitivity, low detection limits, and catalytic activity [14,15,17,20]. However, transition metal hydroxides present some difficulties in terms of their conductivity which depends on the materials being semiconductive and highly insulating. Hence, there is a need to incorporate the carbon nanotubes as conductive supports in order to improve these properties [20]. This work also assessed the peroxidase-like activity of glucose at a glassy carbon electrode appended with a CNT–Co(OH)_2_ composite.

## 2. Materials and Methods

### 2.1. Instruments Y Reagents

The following equipment was used: a Perkin Elmer 100 FT-IR Fourier-transform infrared resonance (FTIR) spectrometer, a LabRAM HR Evolution spectrometer, a FEI Tecnai G2 Spirit TWIN Transmission Electron Microscope (TEM), a Siemens D5005 X-ray diffractometer, a Branson 3800 ultrasonic bath, an electrochemical cell consisting of a Ag/AgCl (3 mol L^−1^) reference electrode, a graphite counter electrode, and a 3 mm diameter glassy carbon working electrode, 1 µm and 0.05 µm alumina powder (CH-Instruments, Inc., Austin, TX, USA.), and a Biologic SP-300 potentiostat, EC-Lab V11.60 software. All solutions were prepared with distilled/deionized water, carbon nanotubes were obtained from NANOCYL^®^NC7000, N,N-dimethylformamide (DMF; CAS 68-12-2) was from Sigma-Aldrich, St. Louis, MO, USA, potassium ferricyanide (K_3_[Fe(CN)_6_]; CAS 13746-66-2), potassium ferrocyanide (K_4_[Fe(CN)_6_]; CAS 14459-95-1), and potassium chloride (KCl; CAS 7447-40-7) were from BDH Chemicals (Dorset, UK), sulfuric acid (H_2_SO_4_; CAS 7664-93-9) and D-glucose (C_6_H_12_O_6_; CAS 50-99-7) were obtained from Fisher Scientific (Waltham, MA USA), ammonium sulfate ((NH_4_)_2_SO_4_; CAS 7783-20-2), sodium citrate dihydrate (Na_3_C_6_H_5_O_7_·2H_2_O; CAS 6132-04-3), cobalt chloride hexahydrate (CoCl_2_·6H_2_O; CAS 7791-13-1), and sodium hydroxide (NaOH; CAS 1310-73-2) were from Sigma-Aldrich.

### 2.2. Structural Characterization

After synthesizing and modifying the composite, various structural characterization techniques were applied, including Fourier Transform Infrared Spectroscopy (FTIR), Raman Spectroscopy, Transmission Electron Microscopy and X-ray diffraction.

### 2.3. Modification of the Glassy Carbon Electrode

#### 2.3.1. Cleaning of the Glassy Carbon Electrode

Mechanical polishing of the electrode was performed using alumina powder of 1 and 0.3 µm for 5 min, followed by rinsing with distilled water. Electrochemical cleaning was carried out in a 5 mL electrochemical cell with three electrodes: a graphite rod as counter electrode, a glassy carbon electrode as working electrode, and a Ag/AgCl (3 mol L^−1^) reference electrode. Cyclic voltammetry was performed at a potential range of 1 to 2 V, with a scan rate of 500 mV s^−1^ for 60 cycles in a 0.1 mol L^−1^ sulfuric acid solution. All solutions in this study were prepared using analytical-grade reagents and deionized water.

#### 2.3.2. Preparation of the fCNT/Co(OH)_2_ Electrode

The fCNTs suspension was prepared by dispersing fCNT in DMF at a concentration of 15 mg mL^−1^ and sonicating it for 2 h. For electrode modification, 10 µL of the fCNT suspension was drop-cast onto the electrode surface, dried at 35 °C for 15 min, and stored in a desiccator until use. The GCE/fCNT electrode was activated in a solution containing 0.45 mol L^−1^ ammonium sulfate and 0.20 mol L^−1^ sodium citrate by performing cyclic voltammetry in a potential range of 0.2 to 0.8 V at a scan rate of 50 mV s^−1^ for 10 cycles. Cobalt electrodeposition was carried out in a solution containing 0.45 mol L^−1^ ammonium sulfate, 0.20 mol L^−1^ sodium citrate, and 10 mmol L^−1^ cobalt chloride (II), using chronoamperometry at −1 V for 3 min, followed by rising with distilled water.

### 2.4. Electrochemical Characterization of the fCNT/Co(OH)_2_ Electrode

The electrochemical characterization of the modified electrodes was conducted by cyclic voltammetry in a 5 mmol L^−1^ potassium ferrocyanide solution with 1 mol L^−1^ potassium chloride at a potential range of 0.2 to 0.8 V, with scan rates ranging from 10 to 120 mV s^−1^. The electrochemical process of the modified electrodes was analyzed using the Randles-Sevick equation (Equation (1))

(1) $$i_{p}=2.69\times 10^{5}\;n^{3/2}\;A\;C\;D^{1/2}\;v^{1/2},$$

where $i_{p}$ (A) is the peak current, A is the electrode area (cm^2^), C is the concentration of the species in solution (mol cm^−3^), v is the scan rate (V s^−1^), and D is the diffusion coefficient (cm^2^ s^−1^).

The electrochemical capacitance of the modified electrodes was obtained by cyclic voltammetry in a 1 mol L^−1^ potassium chloride solution, at potentials ranging from 0 to 0.2 V, following the equation

(2) j=IcA=Cdlv,

where j is the current density (A cm^−2^), $C_{dl}$ is the double layer capacitance (F), and $I_{c}$ is the current (A).

### 2.5. Glucose Detection

Before glucose quantification, its electrochemical behavior was analyzed using cyclic voltammetry, with glucose concentrations ranging from 2.5 to 10.0 mmol L^−1^ on the modified GC/fCNT/Co(OH)_2_ electrode, using 0.1 mol L^−1^ NaOH as the supporting electrolyte, at a potential range of −0.2 to 0.8 V and a scan rate of 50 mV s^−1^.

To assess the sensitivity and linearity of the glucose response on the modified electrode, chronoamperometry was conducted at potentials of 0.45 V, 0.50 V, 0.55 V, and 0.60 V using a 0.1 M NaOH solution as the supporting electrolyte. With constant stirring and successive additions of 20 µL of a 12.5 mM glucose stock solution every 10 s, a calibration plot was obtained in the concentration range of 50 to 190 µmol L^−1^.

For the construction of the calibration curve, measurements were performed in triplicate to evaluate and ensure precision, accuracy, and repeatability. Differences between the means were initially analyzed using a one-way analysis of variance (ANOVA), followed by a Tukey multiple comparison test (*p* < 0.05). Additionally, the limit of detection (LOD) and the limit of quantification (LOQ) were calculated using the following equations, based on IUPAC guidelines [30]

(3) $$LOD=\frac{3.3{\left(1+h_{0}\right)}^{1/2}S_{y}}{b}$$

(4) LOQ=3×LOD

where $h_{0}$ is the leverage for the blank sample, $S_{y}$ is Standard error of the signal, and b is the slope of the regression line (calibration curve) [30].

## 3. Results

### 3.1. Structural Characterization

#### 3.1.1. Transmission Electron Microscopy

**Figure 1:**
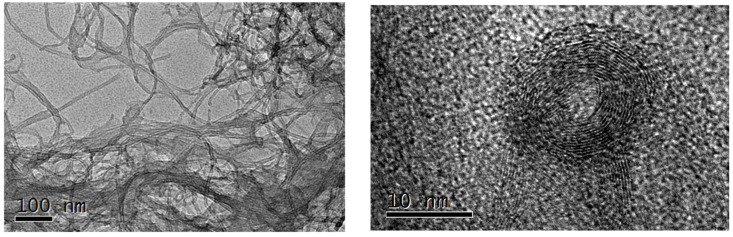
TEM images of unfunctionalized (CNTs).

**Figure 2:**
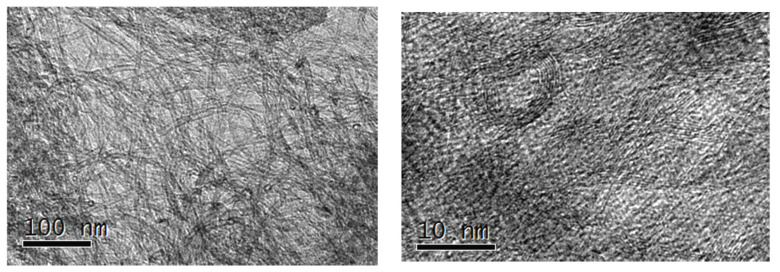
TEM images of the functionalized CNTs (fCNTs).

**Figure 3:**
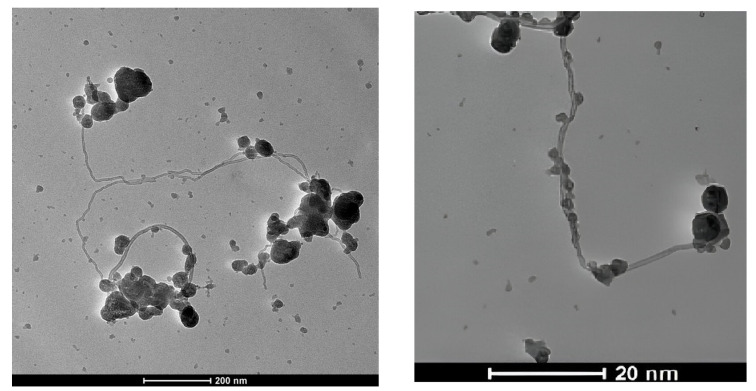
TEM images of the functionalized nanotubes modified with cobalt hydroxide (Co(OH)2-fCNTs).

In Figure 1 and Figure 2, micrographs of unfunctionalized and functionalized CNTs obtained with transmission electron microscope are presented. Functionalized CNTs (fCNTs) have better dispersion and a more defective layer structure than unfunctionalized nanoparticles. Figure 3 suggests that the nanostructured surface morphology exhibits cobalt hydroxide particles (Co(OH)_2_-fCNTs). The particles in question are not uniformly attached to all the fCNTs but are rather seen to be clustering.

#### 3.1.2. Fourier Transform Infrared Spectroscopy

**Figure 4:**
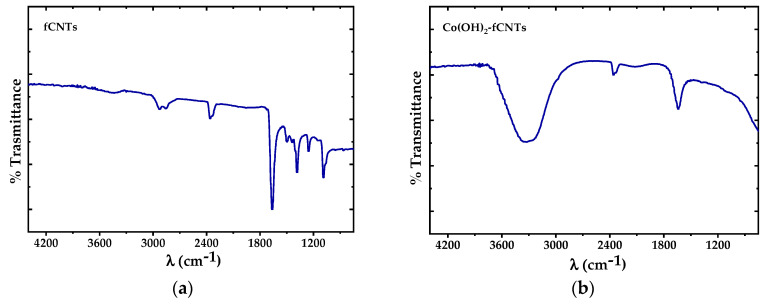
FTIR spectra from 4400 to 1000 cm^−1^ of: (**a**) fCNTs and (**b**) Co(OH)_2_-fCNTs.

FTIR spectra of fCNTs and CNTf/Co(OH)_2_ are presented in Figure 4. In Figure 4a, bands are visible in the region of 1000–1800 cm^−1^ with a main maximum in the 1700 cm^−1^ range corresponding to the carbonyl group (C=O) vibrations. The C=C, C-O-C, and C-H vibrations for CNTs are detectable in the range of 1200–1500 cm^−1^ [31]. In Figure 4b, a band around 3400 cm^−1^ can be seen in the FTIR of the cobalt hydroxide modified fCNTs which is attributed to the stretching of O-H groups of the cobalt hydroxides [27].

#### 3.1.3. Raman Spectroscopy

**Figure 5:**
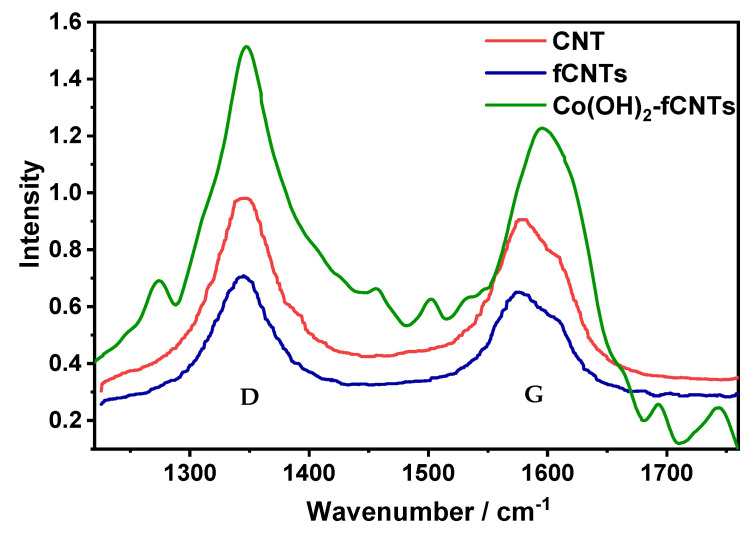
Raman spectra.

In Figure 5, we report the Raman spectra of CNTsf, fCNTs, and Co(OH)_2_-fCNTs. Two archetypal peaks at ≈1340 cm^−1^ and ≈1570 cm^−1^ are called the D-band and G-band, respectively. The D-band is associated with A₁g modes resulting from the graphitic planes, other forms of carbon, and imperfections at the interior surfaces of the nanotubes [32]; the G-band is derived from the C-C oscillations in graphene. However, it can also be noted that, in the spectra of both unmodified and modified CNTs, there is some kind of a shoulder on the G-band at ≈1615 cm^−1^; this is connected to the double resonance from defects or structural disorder [28].

#### 3.1.4. X-Ray Diffraction

**Figure 6:**
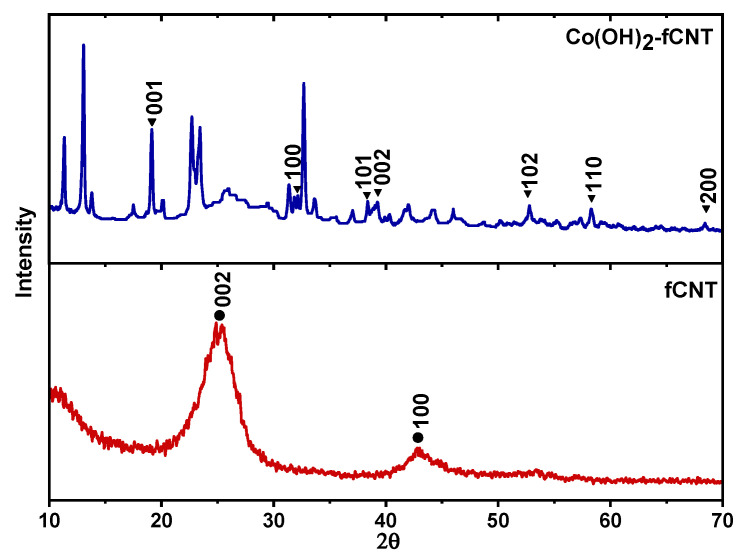
PXRD patterns of Co(OH)_2_-fCNTs and fCNT.

Figure 6 shows diffractograms of fCNT and Co(OH)_2_-fCNT. The X-ray pattern, in red color, of the reduced graphene oxide shows the peak at around 2θ = 25°. In contrast, the PXRD pattern, in blue color, indicates the existence of a structure related to Co(OH)_2_ on the carbon nanotubes.

### 3.2. Electrochemical Characterization of the fCNT/Co(OH)_2_ Electrode

**Figure 7:**
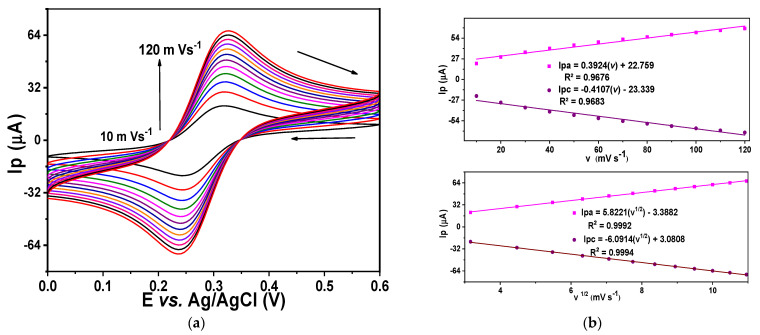
Electrochemical characterization of glassy carbon (GC). (**a**) Cyclic voltammogram at different rates in a solution K_3_Fe(CN)_6_/K_4_Fe(CN)_6_ 5.0 mM + KCl 0.1 M, (**b**) linearization of anodic and cathodic peak current versus velocity, and linearization of anodic and cathodic current versus the square root of velocity.

**Figure 8:**
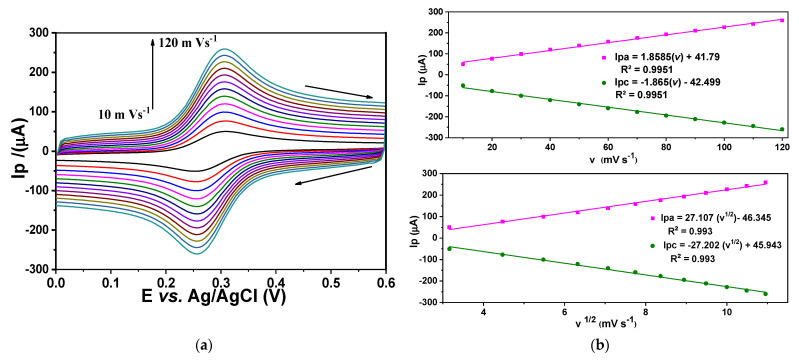
Electrochemical characterization of the modified fCNTs/GC electrode. (**a**) Cyclic voltammogram at different rates in a solution K_3_Fe(CN)_6_/K_4_Fe(CN)_6_ 5.0 mM + KCl 0.1 M, (**b**) linearization of anodic and cathodic peak current versus velocity, and linearization of anodic and cathodic current versus the square root of velocity.

**Figure 9:**
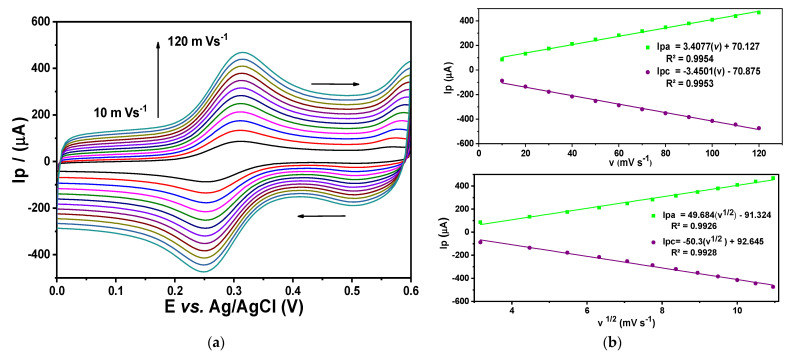
Electrochemical characterization of the modified Co(OH)_2_-fCNTs/GC. (**a**) Cyclic voltammogram at different rates in a solution K_3_Fe(CN)_6_/K_4_Fe(CN)_6_ 5.0 mM + KCl 0.1 M, (**b**) linearization of anodic and cathodic peak current versus velocity, and linearization of anodic and cathodic current versus the square root of velocity.

Electrochemical characterization was performed to obtain parameters such as the electroactive area, using an ideal redox pair (potassium ferricyanide and potassium ferrocyanide), and capacitance for each evaluated electrode: glassy carbon (Figure 7) fCNTs/GC modified electrodes (Figure 8) and Co (OH)_2_ − fCNTs /GC modified electrodes (Figure 9).

The electroactive areas obtained with Equation (1) are collected in Table 1. After modifying the electrode with the nanostructured systems, the electrode area at GC increased notably, especially in the Co(OH)_2_-fCNTs/GC modified electrode.

**Figure 10:**
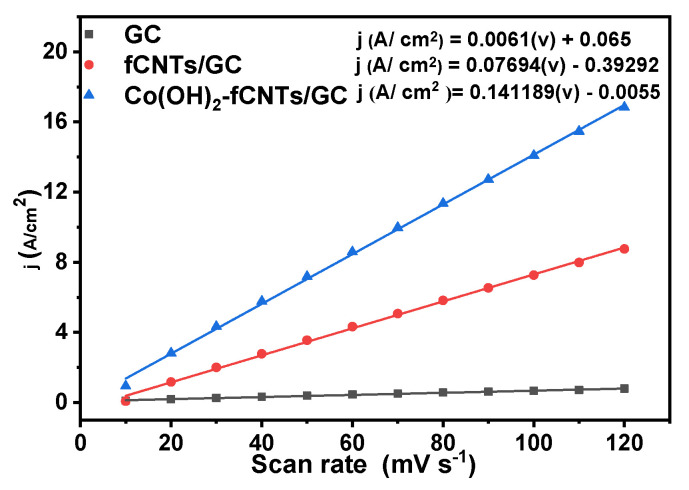
Linear regression of background current density against sweep speed (capacitance).

The plots of linear regression of background current density as a function of scan rate for the GC electrode, fCNTs/GC electrode, and Co(OH)_2_-fCNTs/GC modified electrode can be seen in Figure 10. From Equation (2), capacitance was calculated to be 0.14189475 F/cm^2^ of the Co(OH)_2_-fCNTs/GC electrode; it was higher than those of the other two electrodes (Table 1).

### 3.3. Glucose Detection

**Figure 11:**
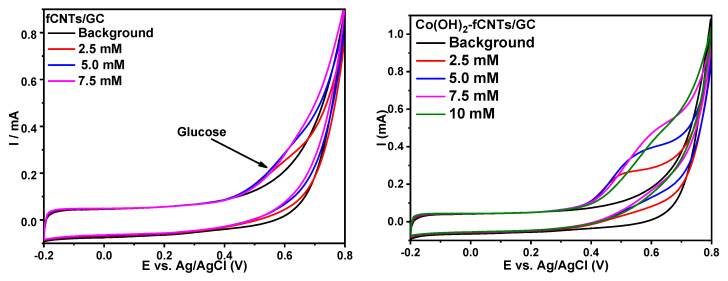
Cyclic voltammograms of the glucose response to different concentrations in NaOH solution 0.1 mol L^−1^ at the modified electrodes.

**Figure 12:**
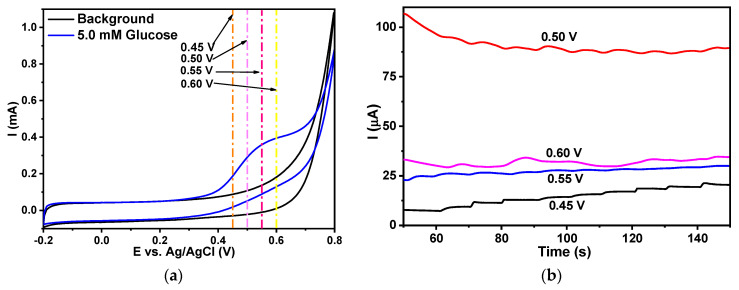
(**a**) Cyclic voltammogram of the glucose response on the modified electrode Co(OH)_2_-fCNTs/GC, (**b**) chronoamperometry response of glucose to different potentials at the modified electrode Co(OH)_2_-fCNTs/GC.

**Figure 13:**
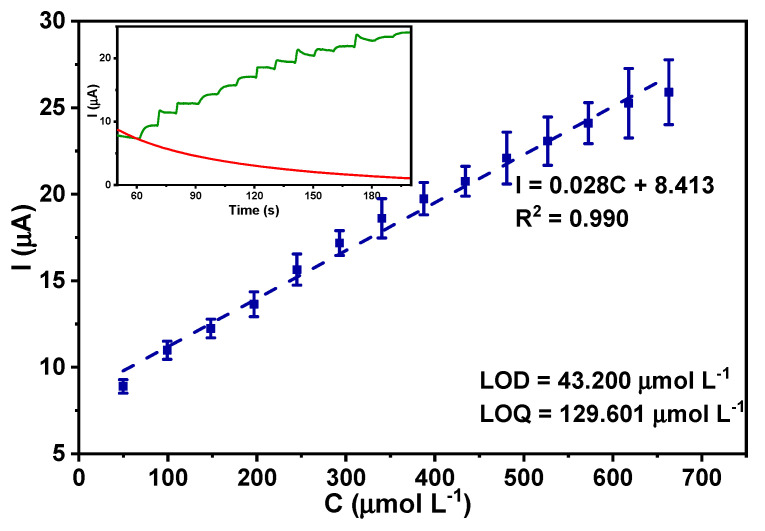
Calibration curve of glucose at modified electrode Co(OH)_2_-fCNTs/GC, NaOH 0.1 mol L^−1^. Insert: chronoamperometry detection.

The electrochemical response of glucose was examined in all the nanostructured systems and the greatest response was observed on the Co(OH)_2_-fCNTs/GC electrode, where the glucose oxidation peak potential was around 0.6 V, as represented in Figure 11. Experimental data are presented in Figure 12 for glucose on the Co(OH)_2_-fCNTs modified electrode. Cyclic voltammetry of glucose (5.0 mM) has been carried out in 0.1 M NaOH solution as a supporting electrolyte as shown in Figure 12a where the process of glucose oxidation starts at 0.35 V. Four potentials in the range of 0.45 V to 0.60 V were chosen for further chronoamperometric studies based on which 0.45 V showed the most distinct glucose response (Figure 12b). This potential was therefore chosen for the calibration curve (Figure 13).

For the calibration curve (Figure 13), three replicates were conducted on the modified electrode under the same condition using chronoamperometry at a potential of 0.45 V and the results included the following: linear range was obtained as 50 to 700 µM L^−1^, linear correlation coefficient was obtained as 0.990, sensitivity as 0.028 µA/µM, and detection limit as 43.200 µM L^−1^.

To compare the replicates, the mean, standard deviation, and coefficient of variation were determined using a simple linear regression model for the three replicates at each concentration point. The calculated values were not more than 10%, proving that the levels of covariation we had estimated were acceptable analytically.

Table 2 shows the parameters obtained from the linear regression analysis of the glucose calibration curve using the Co(OH)_2_-fCNTs/GCE electrode.

The experimental data were evaluated using a simple linear regression model, yielding an R^2^ value of 0.099, which corroborates the linear fit of the data. The sensitivity, limit of detection (LOD), and limit of quantification (LOQ) values obtained are detailed in Table 2. Table 3 also presents the analysis of variance (ANOVA), showing the F-value (calculated from the experimental data) and the critical F-value (tabulated). A comparison reveals that the calculated F-value is greater than the tabulated F-value, confirming a statistically significant linear correlation [33].

## 4. Discussion

Carbon nanotube (CNT) has a cylindrical structure with great features for electronic, mechanical, and thermal applications including in energy storage, catalysis, and sensors. However, CNTs appear to be hydrophobic and chemically stable to some extent and have restricted interactions with other chemical species, which degrades their efficiency in some applications [13]. To overcome these limitations, the CNTs can be functionalized by having active species grafted onto its surface; introducing active sites onto the CNTs surface through Co(OH)_2_ is expected to improve the ability of CNTs to adsorb and undergo suitable reactions with certain analytes, which can augment the efficiency of CNTs [25].

It can be seen in the TEM images of CNTs, fCNTs, and Co(OH)_2_-fCNTs presented in Figure 1, Figure 2 and Figure 3, respectively, that CNTs have a tubular and an entangled structure. They prefer to interact with one another, possibly because the surfaces of most CNTs are electrically terminated and are not compatible with functional groups that would hinder this interaction. The fCNTs are more dispersed and do not interact and aggregate with each other; in Figure 2, the layers of carbon are not as visible and sharp as in the un functionalized CNTs, indicating defects or disruptions of the crystalline surfaces due to functionalization [25,27]. The morphology of the Co(OH)_2_-fCNTs is presented in Figure 3, which reveals that Co(OH)_2_ particles are uniformly distributed on the fCNTs surface. Sometimes it is seen that Co(OH)_2_ particles accumulate with the other particles they already formed, instead of distributing themselves evenly, thereby forming clusters [20].

We can distinguish significant differences in the shapes of the FTIR spectra in Figure 4 for fCNTs and Co(OH)_2_-fCNTs. In the fCNTs spectrum, a well-defined peak near 1700 cm^−1^ associated with the carbonyl (C=O) stretching mode is apparent [25,27,31] that suggests oxygenated function on the nanotube surface, other peaks between 1500 and 1200 cm^−1^ are assigned to C-O and C=C bonds. However, the spectrum of the fCNTs with the presence of Co(OH)_2_ has an obviously resolved peak at about 3400 cm^−1^, belonging to the hydroxyl (O-H) stretching vibrations [29,31,34].

By analyzing the Raman spectra of CNTs, fCNTs, and Co(OH)_2_-fCNTs (Figure 5), the variation of intensity of D and G bands is noted. I_(D/G) is used to measure the quality of material which is under test [27,32]. The I_(D/G) ratios, which are 1.08, 1.09, and 1.24, were recorded for CNTs, fCNTs, and Co(OH)_2_-fCNTs, respectively, indicating that I_(D/G) increases with the functionalization and modification of the carbon nanotubes, probably due to the appearance of new defects on the walls of CNTs each time they are treated [28,35,36].

Figure 6 shows diffractograms of fCNT and Co(OH)_2_-fCNT. The X-ray pattern, in red color, of the reduced graphene oxide shows the peak at around 2θ = 25°, which is assigned to the (002) plane that means ordered graphitic content has been present. Additionally, without a marked peak of graphite at 26° (2θ), the diffractogram shows a less distinct peak at 42° (2θ), which is characteristic of carbon nanotubes [25,37]. In contrast, the PXRD pattern, in blue color, indicates the existence of a structure related to Co(OH)_2_ on the carbon nanotubes: the main diffraction angles are 19.15° and 32.17°depending on the Miller indexation (001), 38.38 and 39.25° (101), 52.79°, 58.32° (102), and 68.43° (200) according to the cobalt hydroxide reference card (JCPDS 00-154-8810) [38].

The performance of the electrodes was characterized in terms of electrochemical process using the cyclic voltammetry and the Randles–Sevick equation (Equation (1)). These are illustrated in Figure 7, Figure 8 and Figure 9 as voltammograms of each electrode against the ideal redox couple [Fe(CN)₆]^3−^/[Fe(CN)₆]^4−^; the oxidation and reduction peaks for these reactions are present [25]. The figures in Table 3 indicate a significant enhancement of electroactive area and capacitance of the modified electrodes over the unmodified GC electrode. The electroactive area rose in the fCNTs/GCE electrode and was even more massive in the Co(OH)_2_-fCNTs/GCE electrode, implying that these changes enhance surface access and augment contact area [26]. Furthermore, the capacitive current of the Co(OH)_2_-fCNTs/GCE electrode is observed at higher current density in comparison with the other electrodes due to increments in the electron transfer process from the functionalized CNTs with cobalt hydroxides. Greater capacitance and area are desirable for a sensor application due to the capability of boosting sensitivity and analyzing different species in low concentration.

The electrocatalytic properties of fCNTs/GCE and Co(OH)_2_-fCNTs/GCE in interaction with glucose were investigated by cyclic voltammetry in 0.1 mol L^−1^ NaOH as the supporting electrolyte. The most enhanced response for glucose oxidation was determined by employing the Co(OH)_2_-fCNTs modified electrode; they showed well-defined peaks as the concentration of glucose increased at about 0.6 V (Ag/AgCl), while the fCNTs/GCE electrode did not give a defined peak (Figure 11). The electrocatalytic oxidation mechanism of glucose with the Co^4+^/Co^3+^ redox pair from Co(OH)_2_ has been described according to the following reactions [20]:

(5) $${Co(OH)}_{2}+{OH}^{-}\;\to \;CoOOH+H_{2}O+e^{-}$$

(6) $$CoOOH+{OH}^{-}\to \;{CoO}_{2}+H_{2}O+e^{-}$$

(7) $${CoO}_{2}+C_{6}H_{12}O_{6}(Glucose)\;\to \;2CoOOH+C_{6}H_{10}O_{6}(Gluconolactone)$$

With these results it is concluded that there is a positive change in the sensitivity of the fCNTs/GCE electrode due [12,13,14,17,18,19,20,21,22] to modification with Co(OH)_2_. For this reason, and because glucose does not require enzymes to be oxidized, the application of metal oxides and hydroxides has grown in the detection of glucose. In the case of glucose detection, the use of Co(OH)_2_ is already evident in prior research [20]. Here, the authors suggest that Co(OH)_2_ is produced from the electrodeposition solution containing cobalt chloride, ammonium sulfate, and sodium citrate. In this bath, cobalt chloride is used as an active material for cobalt supply, sodium citrate for improving the polarization in electrodeposition process, and ammonium sulfate for providing an alkaline environment. Ammonium ions can immobilize the diffusion of cobalt hydroxide in the surface-active region, whereas the sulfate groups can promote cobalt adsorption. This process can be described by the following reactions:

(8) $${NH}_{4}^{+}+{2H}_{2}O\;\to \;{NH}_{3}\cdot H_{2}O+H_{3}O^{+}$$

(9) $${NH}_{3}\cdot H_{2}O\to \;{NH}_{4}^{+}+{OH}^{-}$$

(10) $${Co}^{2+}+{2OH}^{-}\;\to \;{Co(OH)}_{2}$$

After confirming the oxidation signal of glucose by cyclic voltammetry, four potentials were chosen to obtain the steady state amperometric response of glucose. As it can be seen in the current response of Figure 13 (insert), in relation to the successive addition of glucose, the best potential for its determination was at 0.45 V. Sensitivity was obtained by plotting the chronoamperometric response of glucose and achieved a calibration curve in the linear range of 50 to 100 µmol L^−1^, with a linear equation of I (µA) = 0.028C (µmol L^−1^) + 8.413 and a correlation coefficient of 0.990 and is represented by Figure 13. The limit of detection (LOD) and limit of quantification (LOQ) were calculated using Equations (3) and (4), yielding LOD and LOQ of 43.200 µmol L^−1^ and 129.601 µmol L^−1^, respectively. Hence, it is concluded that employing the Co(OH)_2_-fCNTs modified glassy carbon electrode as a fabricated sensor for detecting glucose is an effective approach and an alternative to using the enzymatic sensors.

**Figure 14:**
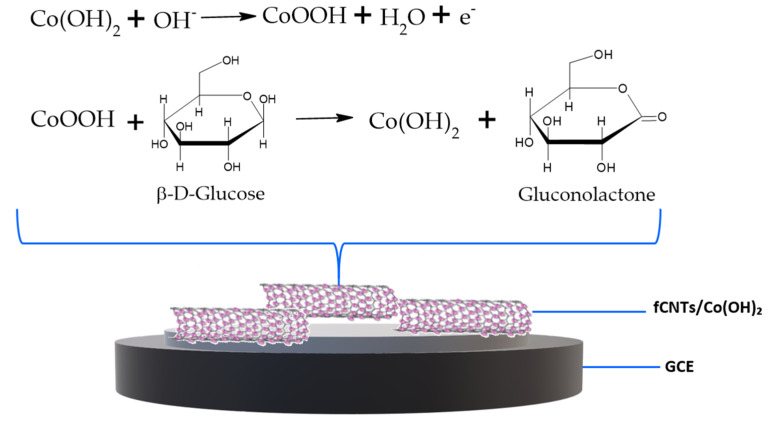
Possible mechanism of glucose electro-oxidation on Co(OH)_2_-fCNTs/GCE.

Cobalt in aqueous solutions exists as a divalent ion with octahedral coordination. However, when ammonium ions from ammonium sulfate are added, these molecules can replace water in the octahedral coordination of cobalt. This phenomenon depends on the ammonium concentration and pH [39]. At pH values between 0 and 7 and at high potential values, cobalt species predominantly exist as the complex Co(H_2_O)₆^2+^ [39,40]. In the pH range of 7–11, ammonium-cobalt complexes form, depending on the number of ammonium ligands ((NH_4_)^+^) and the increasing pH. Finally, at pH values above 11, as studied in the present work, cobalt hydroxide formation is favored [39,41]. A possible mechanism for glucose detection on the fCNTs/Co(OH)_2_/GCE modified electrode is presented in Figure 14, where Co(OH)_2_ is able to transform glucose into gluconolactone. In this mechanism, Co(OH)_2_ undergoes oxidation due to the presence of hydroxyl ions (OH^−^), leading to the formation of cobalt oxyhydroxide (CoOOH). Once formed, CoOOH reacts with glucose, producing gluconolactone [42,43]. Carbon nanotubes, due to their high conductivity and large surface area, facilitate electron transfer; additionally, when doped with Co(OH)_2_, they form a stable and efficient system for glucose electro-oxidation [44].

Table 4 compares the results of the present study with those reported in the literature. The electrode developed demonstrates good electrocatalytic performance; however, the most noteworthy aspect of this work is that the electrode modification through electrodeposition allows the optimization of appropriate reagent proportions. This process enables the formation of a nanostructured system of metal oxides and hydroxides with a more uniform and stable surface.

Non-enzymatic electrochemical sensors for glucose detection address the instability and low sensitivity of enzymatic sensors [45]. Electrochemical modification provides greater control over the growth of nanoparticles on the electrode surface, resulting in a surface with more homogeneous active sites [46]. Another advantage of in situ modification is the reduced use of reagents compared to synthesis methods such as precipitation, sol-gel, hydrothermal combustion, solvothermal processes, etc. [47,48], thereby optimizing laboratory resources and reducing waste generation.

Currently, the mechanisms for developing non-enzymatic electrodes for biomolecule detection using transition metals are not yet fully understood, which can hinder the production and commercialization of these sensors [49]. Future perspectives should focus on the miniaturization of devices, thus reducing application limitations and production costs. Today, to achieve more stable and marketable devices, 3D printing techniques have been implemented [50], involving materials such as polymers or resins sensitive to biomolecules like glucose [51,52,53].

**Table 4:** Comparison of the results of the present study with those reported in the literature.

Electrode (*)	Electrolyte	Sensitive (µA/µM^−1^cm^−2^)	Linear Range (µmol L^−1^)	Detection Limit (µmol L^−1^)	Technique	Reference
Pd-ZnO NRs/GCE	PBS, 0.1 M	0.640	0.0–1.0	0.300	DPV	[54]
MOF-74(Ni)/GCE	KOH, 0.05 M	1.290	10–4000	4.700	I-t plot	[55]
SBHyd-MWCNTs/GC	KOH, 0.1 M	1.100	1000–5000	0.090	I-t plot	[56]
Ag@TiO_2_@ZIF-67/GCE	NaOH, 0.1 M	0.788	48–1000	0.990	I-t plot	[57]
CuO/rGOg/CNF/GCE	NaOH, 0.1 M	0.630	1000–5300	0.100	I-t plot	[58]
NiCoP/Ti	NaOH, 0.1 M	14,856	1000–7000	0.130	I-t plot	[59]
Ni-MOF	NaOH, 0.1 M	14,845	40–2000	0.085	I-t plot	[60]
Au-N-GQDs/GCE	NaOH, 0.1 M	-	0.01–0.05	3.310	ECL	[61]
Co_3_O_4_/NiCo_2_O_4_ DSNCs@G/GCE	NaOH, 0.1 M	304	10–3520	0.384	I-t plot	[62]
Co_3_NNW/TM	NaOH, 0.1 M	3325.6	1–2500	0.050	I-t plot	[42]
(fCNTs)/Co(OH)_2_/GCE	NaOH, 0.1 M	0.028	50–700	43.200	I-t plot	Current study

* Palladium-doped ZnO Nanostructures/Glassy carbon electrode (Pd-ZnO NRs /GCE), flower-like Ni(II)-based metal–organic framework-decorated Ag nanoparticles/glassy carbon electrode (MOF-74(Ni)/GCE), multiwalled carbon nanotubes-based copper (II) Schiff base complex/ glassy carbon electrode (SBHyd-MWCNTs/GCE), metal-based electrode metal organic framework modified with silver nanoparticles and titanium dioxide/glassy carbon electrode ((Ag@TiO_2_@ZIF-67/GCE), copper oxide nanoneedles, graden and carbon nanofibers/glassy carbon electrode (CuO/rGOg/CNF/GCE), ternary NiCoP nanosheet array on a Ti mesh (NiCoP/Ti), a Ni-based metal–organic framework nanosheet array on Ni foam (Ni-MOF), nitrogen-doped graphene quantum dots coated with gold nanoparticles/glassy carbon electrode (Au-N-GQDs/GCE), graphene-wrapped porous Co3O4/NiCo2O4 double-shelled nanocages/glassy carbon electrode (Co3O4/NiCo2O4 DSNCs@G/GCE), cobalt-nitride nanowire array (Co3NNW/TM), nanoporous cobalt-oxide nanowires/ glassy carbon electrode (Co3O4NW/GCE).

## 5. Conclusions

It is shown in this work that a non-enzymatic glucose sensor, with the use of fCNTs-Co(OH)_2_, can be an efficient solution for glucose detection compared to enzymatic methods. The interaction of Co(OH)_2_ with fCNTs greatly improves the electrochemical active surface area and the capacitance of the electrode that yields a higher sensitivity and an almost plateau for the electrochemical response to glucose altogether. From the TEM, Raman, and FTIR analysis, the structural characterization of Co(OH)_2_ particles and fCNTs demonstrated the appropriate incorporation of Co(OH)_2_ particles onto fCNTs surface.

These results combined to give the linear response within the concentration range of the glucose from 50 to 700 µmol L^−1^, with the LOD at 43.200 µmol L^−1^, LOQ at 129.601 µmol L^−1^, and sensitivity of 0.028 µA/µmol L^−1^. These characteristics suggest that the composite formed by Co(OH)_2_-fCNTs behaves as a good catalyst and improves the electrochemical activity of the sensor, thus allowing accurate glucose sensing without involving enzymes.

## Figures and Tables

**Table 1:** Electrochemical parameters obtained from the modified electrodes.

Electrode	Area (cm^2^)	Capacitance (F cm^−2^)
GCE	2.3774 × 10^−6^	0.00605267
fCNTs/GCE	1.403 × 10^−5^	0.07693774
Co(OH)_2_-fCNTs/GCE	1.4035 × 10^−5^	0.14189475

**Table 2:** Parameters obtained from the linear regression analysis.

Regression Statistics	
Multiple correlation coefficient	0.995
Determination coefficient R	0.990
Adjusted R	0.990
Typical error	0.549
Data number	14
Intercept	8.413
Slope	0.028
Detection Limit	43.200
Quantitation Limit	129.601

**Table 3:** Analysis of variance.

	DOF	Sum of Squares	Mean Square	F	Critical Value of F
Regression	1	389.744	389.744	1291.601	1.37801 × 10^13^
Residual	12	3.621	0.302		
Total	13	393.365			

## Data Availability

The raw data supporting the conclusions of this article will be made available by the authors on request.
